# Stem cell phenotype predicts therapeutic response in glioblastomas with MGMT promoter methylation

**DOI:** 10.1186/s40478-022-01459-9

**Published:** 2022-11-04

**Authors:** Nelli S. Lakis, Alexander S. Brodsky, Galina Karashchuk, Amanda J. Audesse, Dongfang Yang, Ashlee Sturtevant, Kara Lombardo, Ian Y. Wong, Ashley E. Webb, Douglas C. Anthony

**Affiliations:** 1 Department of Pathology, Kansas University Medical Center, Kansas City, KS USA; 2 Department of Pathology and Laboratory Medicine, Lifespan Academic Medical Center, Providence, Rhode Island USA; 3 Department of Pathology and Laboratory Medicine, Warren Alpert Medical School of Brown University, Providence, Rhode Island USA; 4 Center for Computational Molecular Biology, Brown University, Providence, Rhode Island USA; 5 Department of Molecular Biology, Cell Biology, and Biochemistry, Brown University, Providence, Rhode Island USA; 6 School of Engineering, Brown University, Providence, Rhode Island USA; 7 Center on Biology of Aging, Brown University, Providence, Rhode Island USA; 8 Carney Institute for Brain Science, Brown University, Providence, Rhode Island USA; 9 Department of Neurology, Warren Alpert Medical School of Brown University, Providence, Rhode Island USA

**Keywords:** Glioblastoma, Cancer stem cells, SOX2, CD133, MGMT

## Abstract

A growing body of evidence supports the presence of a population of cells in glioblastoma (GBM) with a stem cell-like phenotype which shares certain biological markers with adult neural stem cells, including expression of SOX2, CD133 (PROM1), and NES (nestin). This study was designed to determine the relationship between the expression of these stem cell markers and the clinical outcome in GBM patients. We quantified the intensity of expression of the proteins CD133 and SOX2 by immunohistochemistry (IHC) in a cohort of 86 patients with IDH-wildtype GBM, and evaluated patient outcomes using Kaplan–Meier and Cox proportional hazards analysis. In our patients, *MGMT* promoter methylation status and age were predictors of overall survival and progression free survival. The levels of SOX2 and CD133 were not associated with outcome in univariate analysis; however, stratification of tumors based on low or high levels of CD133 or SOX2 expression revealed that *MGMT* methylation was a predictor of progression-free survival and overall survival only for tumors with high levels of expression of CD133 or SOX2. Tumors with low levels of expression of CD133 or SOX2 did not show any relationship between *MGMT* methylation and survival. This relationship between *MGMT* and stem cell markers was confirmed in a second patient cohort, the TCGA dataset. Our results show that stratification of GBM by the level of expression of CD133 and SOX2 improved the prognostic power of *MGMT* promoter methylation status, identifying a low-expressing group in which the clinical outcome is not associated with *MGMT* promoter methylation status, and a high-expressing group in which the outcome was strongly associated with *MGMT* promoter methylation status. These findings support the concept that the presence of a high stem cell phenotype in GBM, as marked by expression of SOX2 or CD133, may be associated with the clinical response to treatment.

**Supplementary Information:**

The online version contains supplementary material available at 10.1186/s40478-022-01459-9.

## Introduction

Glioblastoma (GBM) is the most common malignant primary tumor of the central nervous system (CNS) [22]. GBMs account for 47% of primary malignant brain tumors and are more common in older adults. Even with the most current treatment modalities, including surgery, radiation and temozolomide (TMZ) chemotherapy, only 5% of patients survive beyond five years after diagnosis [28]. Better tools are needed to identify which patients will respond to therapy and achieve longer survivals. Biomarkers offer a promising approach for improving prognostication and guiding personalized therapies to prevent late tumor recurrence. The late tumor recurrences after initial clinical response of GBM has led to a search for relationships between the presence of cancer stem cells (CSC) and the resistance of GBM to therapy [6].

GBMs are characterized by the presence of a population of treatment-resistant cancer cells which have “stem-like” features. These cancer stem cells (CSCs) are thought to be responsible for recurrence of tumor after therapy [9, 35]. CSCs are functionally defined as cells that contribute to tumor initiation and therapeutic resistance [20]. However, the definition of a cancer “stem cell”, or a specific marker to identify it, remains fluid [20].

The CSC hypothesis predicts that CSCs are a small subpopulation of cells in tumors and yet many CSC markers, including CD133 and SOX2, are often highly expressed in GBMs. Deregulation of embryonic stem cell expression programs is prevalent in many GBMs [7, 39], which may explain the observed high percentage of cancer cells expressing putative CSC markers. Among the individual proteins that have been identified as potential stem cell markers are SOX2, CD133, NES, Nanog, and Oct4, based on the observation of their expression in embryos and in tumors [4, 10]. We chose to focus on three putative stem cell markers, SOX2, NES, and CD133, as markers of the cancer stem cell phenotype in order to search for heterogeneity of expression within tumors, interpatient variability in expression patterns, and any possible associations with outcomes or other clinicopathological characteristics.

SOX2 is a transcription factor that promotes proliferation of stem cells, but impedes cell-type commitment [30]. It is expressed even after stem cells have differentiated [8–10, 14], and is frequently highly expressed in GBMs [4, 16]. It is more highly expressed than some other putative CSC markers, such as OCT4 [8, 9]. SOX2 has been associated with a GBM “proneural” molecular signature, along with other regulators of neural stem/progenitor cell fate [37]. SOX2 is a major index of “stemness” in neural development, and the miR-21/*SOX2* axis has prognostic importance in GBM [32].

CD133 is a surface glycoprotein expressed by neural stem cells encoded by *PROM1*, and is a marker of therapy-resistant, tumorigenic cells in cancer stem cell models [12, 13]. Consistent with the potential importance of CD133 in GBMs, high levels of *PROM1* transcript levels in GBMs have been associated with shorter survival in some series[26, 33]. Isolation of CD133-positive cells enriches for a subset of cancer cells with higher proliferation rate and increased divergent differentiation, but the transcript level of *PROM1* or the protein level of CD133 is not considered to be a defining marker of the stem cell state [18, 20]. The association between patient overall survival and CD133 protein expression is not firmly established, although the evidence trends toward increased expression being associated with shorter survival [38]. Combinations of additional stem cell markers with CD133, including SOX2 and NES, have been proposed to be associated with higher risk [40]. NES is an intermediate filament protein associated with neural stem cells during brain development, encoded by the gene *NES*, and associated with histologic grade of gliomas [10].

Methylation of the promoter region of the gene encoding O^6^-methylguanine-DNA methyltransferase (*MGMT*) is an epigenetic regulatory mechanism that silences *MGMT* expression. The *MGMT* gene product is involved in repairing DNA damage from temozolomide (TMZ), and the methylation status of the gene promoter predicts therapeutic response to TMZ [17, 24, 34, 41], but not to surgery and radiation alone [17]. Due to its effect on TMZ therapeutic response, *MGMT* promoter methylation status is a strong prognostic predictor of disease progression and overall survival [17], and, with the almost universal use of TMZ in the treatment of GBM at the present time, recent efforts have explored whether additional clinicopathological characteristics may be combined with *MGMT* promoter methylation to improve prognostic predictions [6].

The level of CD133 and SOX2 expression in GBM may be indicative of the presence of a treatment-resistant cancer cell subpopulation. However, neither CD133 nor SOX2 has been consistently associated with outcomes in GBM patients. Because the presence of *MGMT* promoter methylation is associated with increased survival [17], we hypothesized that stem cell markers may identify biological subsets of GBM when studied in combination with *MGMT* promoter methylation status. Given the important roles of SOX2 and CD133 in GBM stemness and plasticity [4, 12, 13, 30], we studied the relationship between the level of expression of these putative stem cell markers and clinical outcomes in patients with GBM to test the hypothesis that GBM stem cells may have a role in response to therapy.

## Materials & methods

### Patient selection

We searched the files of the Department of Surgical Pathology at The Rhode Island and Miriam Hospitals from January 1, 2012 through August 31, 2016 for all cases of “Glioblastoma” or “Gliosarcoma”, and retrieved 270 cases. Eligibility for inclusion required a diagnosis of Glioblastoma, IDH-wildtype, meeting the criteria of the 2021 WHO Classification of Tumors of the Central Nervous System [22]. Inclusion criteria also included the availability of clinical follow-up information, and sufficient tissue remaining in a diagnostic tissue block to create a tissue microarray. Sequential cases were reviewed until 100 cases were identified. If the original block used for diagnosis and immunohistochemistry was no longer available, a second appropriate block was chosen. Two neuropathologists (NSL, DCA) agreed on the diagnoses of glioblastoma, IDH-wildtype for all tumor samples in accordance with the most current World Health Organization classification [22].

### Clinical data

Clinical data for these 100 patients were collected from review of the medical records, which included demographic information, clinical presentation, tumor location, tumor laterality, surgical procedure, surgical resection status (gross total resection, GTR or subtotal), tumor volume, date of MRI- or CT-documented recurrence and/or progression, date of last follow-up, date of overall survival, and treatment with radiation and/or concomitant temozolomide. Data was collected according to protocols approved by the Institutional Review Board of Rhode Island Hospital.

### Pathology data

Pathology data was collected from whole slides, external pathology data, and surgical pathology reports. The data collected included: IDH1-R132H mutation (HistoBioTec clone H09), *MGMT* status (promoter methylation status of O(6)-methylguanine-DNA methyltransferase, measured by PCR after macrodissection and bisulfite treatment), p53 expression (Dako D07 clone), Ki67 expression (Dako, MIB1), 1p19q status (by FISH, when available), and morphologic findings, including any gemistocytes, spindle-cell features, small cell change, oligodendroglial morphology, and/or giant cell change.

### Tissue microarray

Examination of 4-μm thick slides revealed that 98 cases had sufficient tumor remaining in the block to be included in a paraffin-embedded tissue microarray (TMA). 82 cases had three representative tumor regions only, and 16 cases had three representative tumor regions plus a “normal” or “infiltrating tumor” region. The tissue cores were evaluated by two neuropathologists (NSL, DCA) who supervised all TMA construction steps. Each core sample in the TMA was classified as “tumor”, “normal brain”, or “insufficient” (insufficient tissue for assessment, completely necrotic sample, or missing core on the TMA sections). Cores were not included in the tumor final analysis if the core was missing from the slide, severely damaged, and/or did not have a cellular tumor sample. Eight cases were excluded due to lack of any analyzable tumor in the final TMA blocks, three cases were excluded due to IDH1-mutant status (identified on review to have IDH mutation), and two cases were excluded because they were recurrent tumors that did not meet diagnostic histologic criteria of glioblastoma. Using WHO 2021 criteria, eighty-six (*n* = 86) cases were ultimately included in the cohort of IDH-wildtype GBM (in three of these cases, focal areas with biphasic spindle-cell morphology, sometimes referred to as gliosarcoma, were present). Seventy-two cases had *MGMT* promoter methylation status determined at the time of initial diagnosis.

### Immunohistochemistry

Anti-CD133 [Thermo Cat# PA5-38,014, at 1:600 dilution], anti-SOX2 [(clone 20G5) Thermo Cat# MA1-014, at 1:600 dilution], and anti-NES [(clone 10C2) Thermo Cat# MA1-110, at 1:600 dilution] were obtained commercially and used for immunohistochemistry. Four-μm paraffin sections were cut and incubated at 60 °C for 30 min, and the sections were deparaffinized with xylene and rehydrated in graded alcohols (100%, 95%, 70%) and water. Antigen retrieval was performed in citrate buffer with a pressure cooker and microwave for 10 min. Endogenous peroxidase activity was quenched by incubating slides with Dual Endogenous Enzyme Block (Agilent Technologies, Santa Clara, CA). The sections were incubated with the primary antibodies at respective dilutions for one hour in a humidified chamber at 25C, followed by a 30 min incubation with EnVision Dual Link System-HRP (Agilent Technologies, Santa Clara, CA). Antigen–antibody complexes were visualized with peroxidase-based detection systems using diaminobenzidine (DAB) (Agilent Technologies, Santa Clara, CA) as a substrate.

### Immunofluorescence

Since the TMA immunohistochemistry was limited to tissue cores, we also determined the extent of variation of SOX2 expression within larger regions of GBM using immunofluorescence in frozen GBM tumor samples. Using a second set of GBM tumor samples identified in the Tumor Bank of the Rhode Island Hospital (*n* = 16, samples frozen at − 80 °C), a study approved by Institutional Review Board, we used immunofluorescence to localize and quantify the expression of SOX2 in each of these frozen specimens of glioblastoma, IDH-wildtype (WHO grade IV). For immunofluorescence microscopy, specimens were fixed with 4% paraformaldehyde for 24 h at 4 °C, and cryoprotected by incubation in 5% sucrose for 24 h (4 °C). Fixed cryoprotected tissue was stored at − 80 °C.

### Immunofluorescence staining

Fixed and cryoprotected GBM tissues were sectioned at 5 µm on positive-coated slides and rehydrated for 15 min in phosphate-buffered saline, pH 7.4 (PBS). The sections were then post-fixed in 4% paraformaldehyde for 15 min and rinsed in PBS. Non-specific background was blocked by incubation in blocking solution (5% goat serum, 5% donkey serum, 0.3% Triton X-100 in PBS, pH7.4) for 1 h at room temperature. The sections were incubated with anti-SOX2 rat monoclonal antibody (ThermoFisher #14–9811-82, 1:200 dilution) overnight at (+ 4 °C) followed by donkey anti-rat IgG Alexa 488 staining (ThermoFisher #A-21208, 1:1,000 dilution). Slides were mounted with ProLong Glass Antifade Mountant with NucBlue Stain (ThermoFisher #P36981) and cured overnight at room temperature. Immunofluorescence slides were examined with confocal and conventional fluorescence microscopy, using GFP and DAPI filters. For each case, three fields were photographed at 40 × and 120x (60 × 2) magnification, and the images were used for quantitation. The total number of nuclei was identified with NucBlue staining, and total number of cells counted. Individual cells were identified as negative (NucBlue staining only), or positive (SOX2-expressing; Alexa green 488) determined by the presence of a strong nuclear expression of SOX2.

### Scoring of TMAs

Semi-quantitative assessment of the immunohistochemistry results was performed for CD133, SOX2, and NES. For CD133, cytoplasmic and membranous circumferential staining was considered positive. The staining intensity was graded as none (0), weak (1 +), moderate (2 +), or strong (3 +) and was multiplied by the percentage of positive cells, ranging from 0 to 300 (3 × 100%) as the raw score. Four categories were defined as follows from the raw scores: 0 = 0, 1–100 = 1, 101–200 = 2, and 201–300 = 3. For final analysis, CD133 was divided into two categories: scores of 0 or 1 was considered low expression, and scores of 2 or 3 was considered high expression.

SOX2 nuclear staining was considered positive. The staining intensity was graded as none (0), weak (1 +), moderate (2 +), or strong (3 +) and was multiplied by the percentage of positive cells to create a raw score. Four categories were defined as follows from the raw scores: 0 = 0, 1–100 = 1, 101–200 = 2, and 201–300 = 3. Scores of 0 and 1 were considered low SOX2 expression, and scores of 2 and 3 were considered high SOX2 expression.

NES cytoplasmic staining was considered positive. NES, which is expressed in both tumor cells and in neovascularization, was assessed for whether the expression was in tumor cells or in blood vessels in areas of neovascularization. Tumor cell expression intensity was graded as none (0), weak (1 +), moderate (2 +), or strong (3 +) and was multiplied by the percentage of positive cells to create a raw score. Four categories were defined as follows from the raw scores: 0 = 0, 1–100 = 1, 101–200 = 2, and 201–300 = 3. Blood vessel expression of NES was recorded as positive or negative, but was not used in scoring of glioma stem cell expression.

### TCGA RNA-seq analysis

Analysis of bulk tumor RNA-sequencing was performed on 169 GBM tumor samples and 5 normal control samples obtained from The Cancer Genome Atlas Project (TCGA). HTSeq-FPKM files, clinical, and metadata were downloaded using the GDC Data Transfer Tool and GDC data portal from the National Cancer Institute per the data manifest. A log2(FPKM + 1) transformation was then performed on all FPKM values and normal sample controls were removed. GBM tumor samples were then separated based on the expression of individual genes at the median: separating high *SOX2* expressing tumors (above the median) from low *SOX2* expressing tumors (below the median), and high *PROM1* expressing tumors (above the median) from low *PROM1* expressing tumors (below the median). Survival analysis (Log-rank (Mantel-Cox) test) was then performed, where tumors were classified based on high or low expression of *PROM1* or *SOX2* alone and in combination with *MGMT* methylation status. *MGMT* methylation status was mapped to samples by matching the submitter IDs to case IDs where data was available from Brennan et al. [11]. For two-gene correlations, the Betastasis two-gene scatterplot tool (Affymetrix HT HG U133A) and the glioblastoma Rembrandt (GEO GSE108476) dataset were used, with log-2 (FPKM + 1) transformation.

### Statistical analysis

SPSS v22 for MacOS (SPSS, Chicago, IL, USA) was used for all statistical analysis. *P* < 0.05 was considered statistically significant. Overall and progression free survival were estimated by Kaplan–Meier analysis and were compared using the two-sided log-rank test. All tests were two-sided.

## Results

### Patient and clinical information

Among patients treated at Rhode Island Hospital between 2012 and 2016, we identified 86 patients with a new diagnosis of GBM with sufficient tissue for analysis on a tissue microarray (TMA) and available follow-up clinical information. Patients who had small (or needle) biopsies, or those who did not receive their subsequent care in our health care system were not included. Clinical data is summarized in Table 1. The mean and median age at the time of diagnosis was 62 years. A majority of the patients in this cohort was male (60.5%). Gross total resection was achieved in 45% of patients, and all but 5 patients received radiation and TMZ therapy after surgery. Five patients received neither TMZ therapy nor radiation, and their disease rapidly progressed.

**Table 1:** Clinicopathologic characteristics

Clinicopathologic feature		All cases (*n* = 86)
Age at diagnosis	Mean	62.3
	Range	37–83
Gender	Male	52 (60.5%)
	Female	34 (39.5%)
Radiation	Yes	80 (87.9%)
	No	5 (5.5%)
	Not available	1 (1.2%)
TMZ	Yes	80 (93.0%)
	No	5 (5.8%)
	Not available	1 (1.2%)
Laterality	Left	42 (48.8%)
	Right	44 (51.2%)
Lobe	Frontal	30 (34.9%)
	Parietal	13 (15.1%)
	Temporal	39 (45.3%)
	Occipital	3 (3.5%)
	Other site	1 (1.2%)
MGMT status	Methylated	29 (40.3%)
	Unmethylated	39 (54.2%)
	Partial	4 (5.6%)
	Data not available	14 (16.3%)
CD133 (hi)	Score ≥ 1	30 (34.9%)
SOX2 (hi)	Score > 1	53 (61.6%)
NES (hi)	Score > 1	59 (68.6%)
NES blood vessels	Positive	76 (88.4%)

**Fig. 1:**
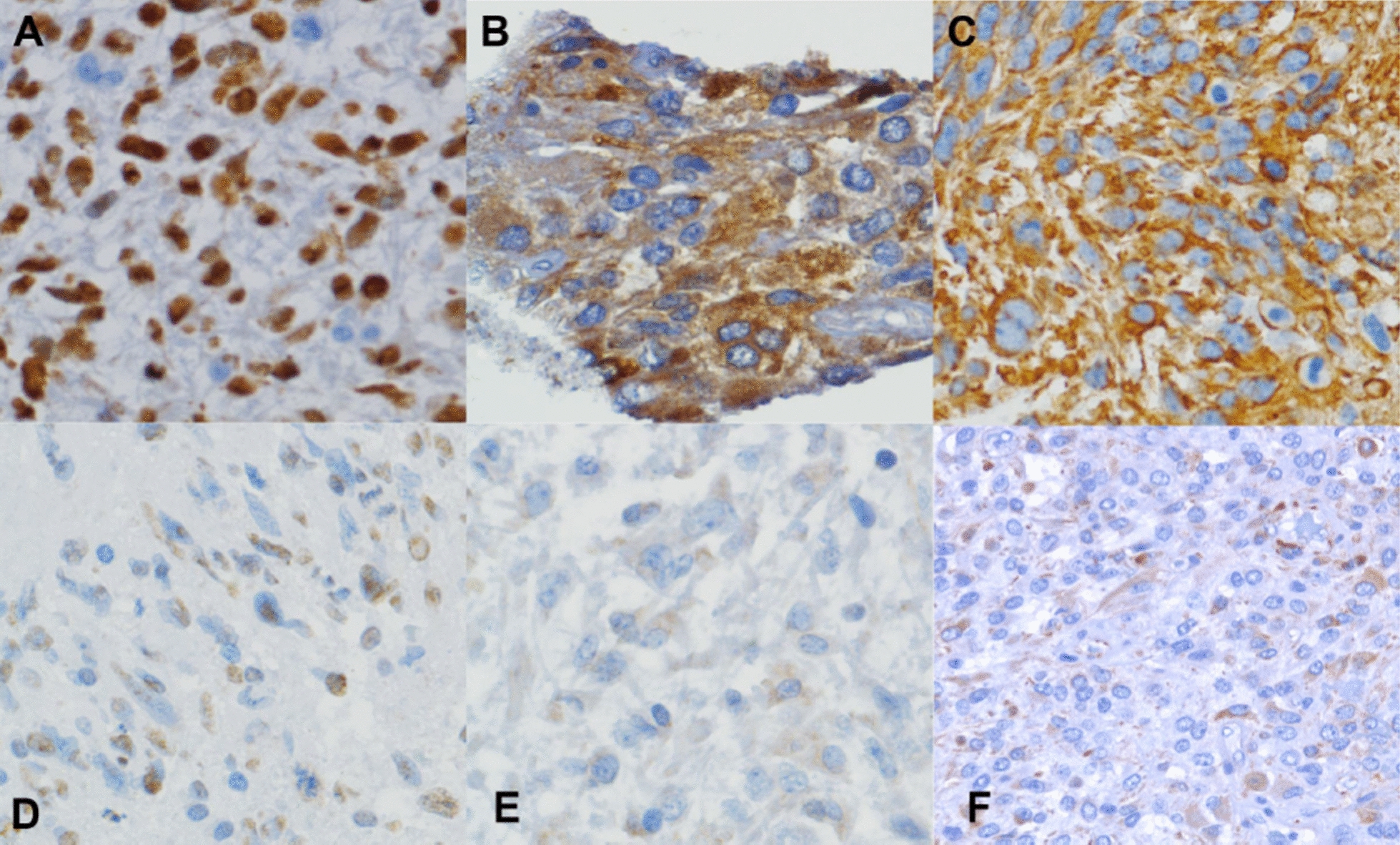
Stem cell marker expression in glioblastoma. Expression of SOX2, CD133, and NES as determined by IHC, was stratified into high and low levels of each stem cell marker. High levels of expression of SOX2 **A**, CD133 **B**, and NES **C** are shown in the upper panels, and low (non-zero) levels of SOX2 **D**, CD133 **E**, and NES **F** are shown in the lower panels

**Fig. 2:**
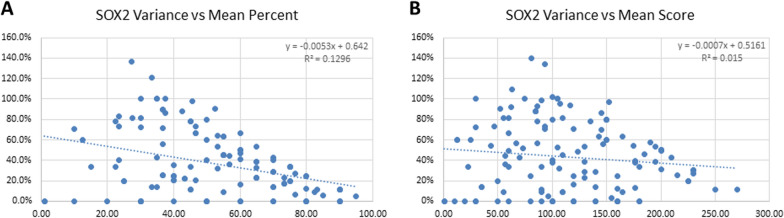
The coefficient of variation (SD/Mean) in percentage of SOX2-positive cells is shown for each patient in the TMA semiquantitative analysis. When the variance is plotted against the mean percent **A** or the mean raw score **B**, there is a higher variance in cases with low levels of SOX2 expression. Using the median cut-off of 50% for percent of SOX2-positive cells in the TMA cores, the mean variance is 48% in the low-expressing group and 26.6% in the high-expressing group (*p* < 0.0005)

There was a wide range of intensity of expression of stem cell markers in GBM (Fig. 1). Individual tumors varied in their expression of each of the stem cell markers (SOX2, CD133, and NES) in both the intensity of expression in individual cells and the percentage of cells expressing the stem cell markers (Table 1). The percent of cells expressing SOX2 (Fig. 2a) ranged from 0% in an individual TMA core to 95%, and the variation (coefficient of variation, calculated as the ratio of standard deviation to mean, expressed as percent) was much higher for cases with a low mean percentage of SOX2-expressing cells. The median percent of cells expressing nuclear SOX2 was 52.5% (Fig. 2a). When cases were separated into high or low percentage of SOX2-expressing GBM (above or below 50% of nuclei positive on average for the case), the mean variation was 48% for samples in cases with a low percentage of SOX2-positive cells, and 26.6% in high-expressing cases (*p* < 0.0005). A similar trend was seen for the variance of SOX2 raw score (the product of mean percentage and intensity score; Fig. 2b).

**Fig. 3:**
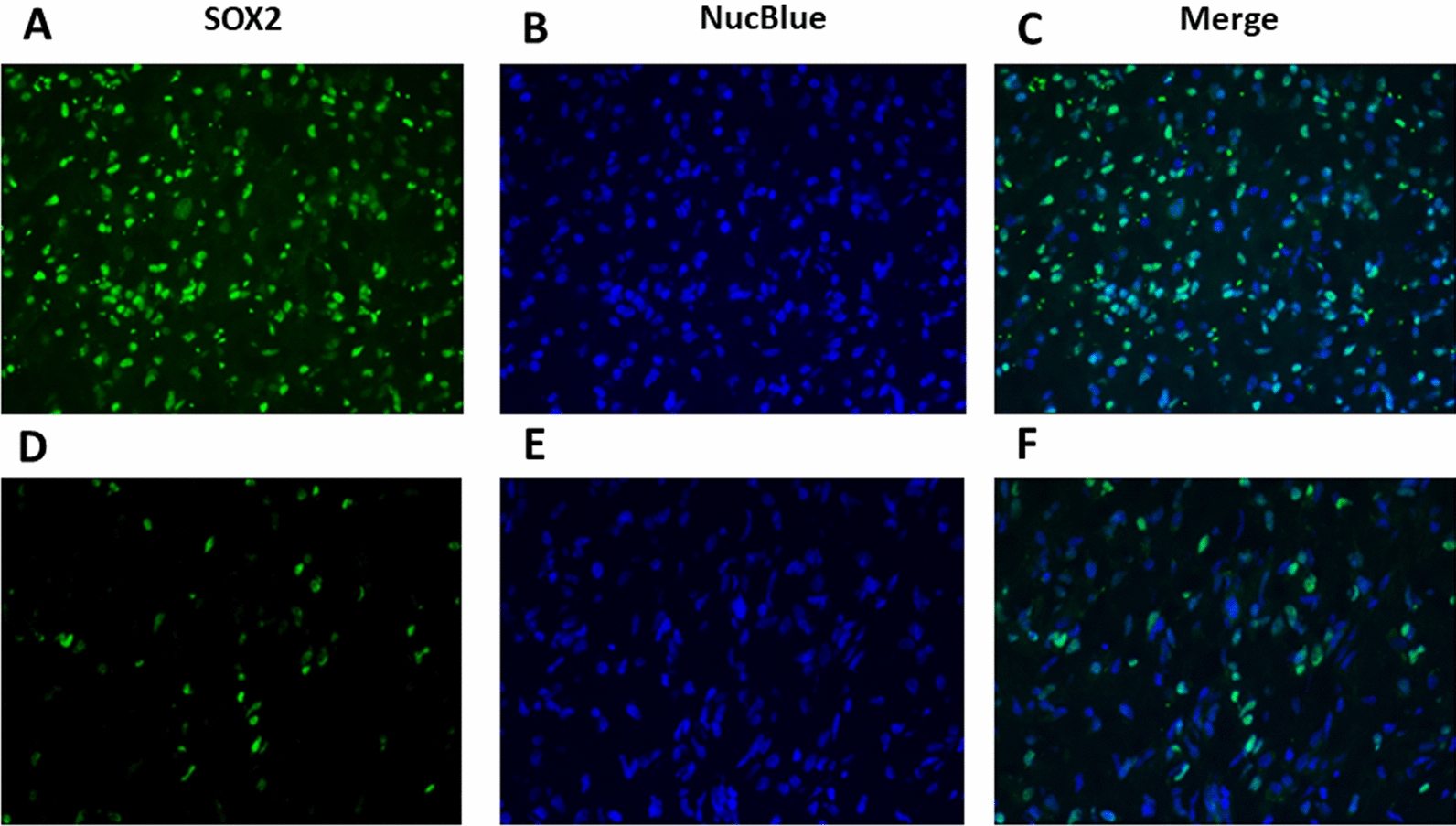
Immunofluorescence for SOX2 in GBM (Alexa Green 488) and total cells (NucBlue). In the top panels **A**–**C**, SOX2 is present in a high percentage of cells and more uniformly distributed within the field (and between fields), than in a low-expressing case (bottom panels, **D-F**), where the percent of SOX2 + cells is lower and varies more across an individual field and between fields. (**A, D** – Alexa Green 488, **B, E** –NucBlue, **C, F** – Overlay)

**Fig. 4:**
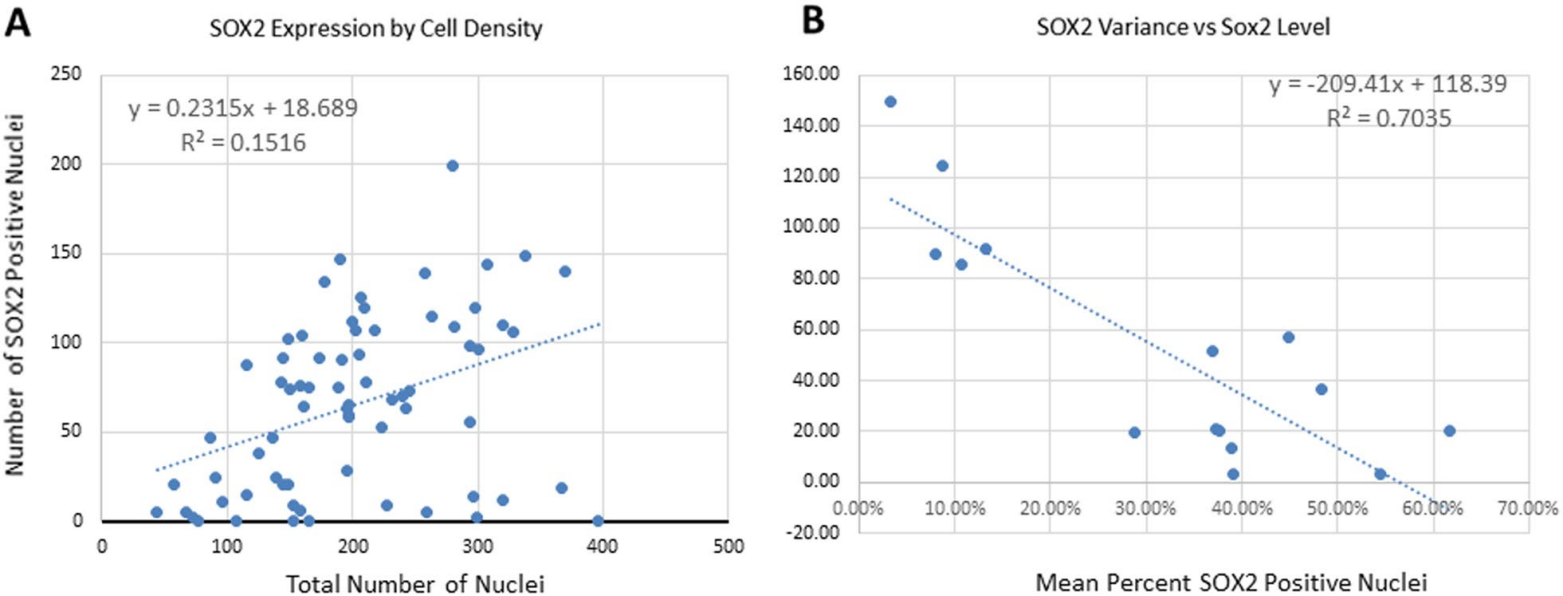
The raw counts of cells in individual fields of all tumors quantified by immunofluorescence are shown **A**, and show a weak tendency for more cellular areas to have more SOX2-positive cells. The coefficient of variation (SD/Mean) for each case **B** shows a strong tendency for higher variability in cases with a low mean percentage of SOX2-positive cells. Using the median cut-off of 30% for percent of SOX2-positive cells by immunofluorescence, the mean variance is 93.5% in the low-expressing group and 25.1% in the high-expressing group (*p* < 0.002)

In a second set of GBM tumor samples (*n* = 16, samples frozen at −80 °C), we used immunofluorescence to study the expression of SOX2 in larger regions of tumor tissue (rather than the cores of the TMA). We observed a wide range of percentage of SOX2 + nuclei among samples, and variability from area to area within the same tumor (Fig. 3, 4). The number of nuclei expressing SOX2 was only weakly related to the total number of cells across all fields (Fig. 4a), with the percentage ranging from 0 to 77.4%, with a median of 32.7% (Fig. 4a). The relationship between variation of SOX2-expressing cells and the mean percentage showed a higher variation in cases with low overall SOX2 expression (Fig. 4b). Using the median cutoff of 30% positive nuclei to separate high-expressing from low expressing tumors, the variation was higher in low-expressing tumors (93.5%) than in high-expressing tumors (25.1%; *p* < 0.002). In both high-expressing tumors and low-expressing tumors, the distribution of SOX2 + cells was not uniform, but occurred in clusters.

**Fig. 5:**
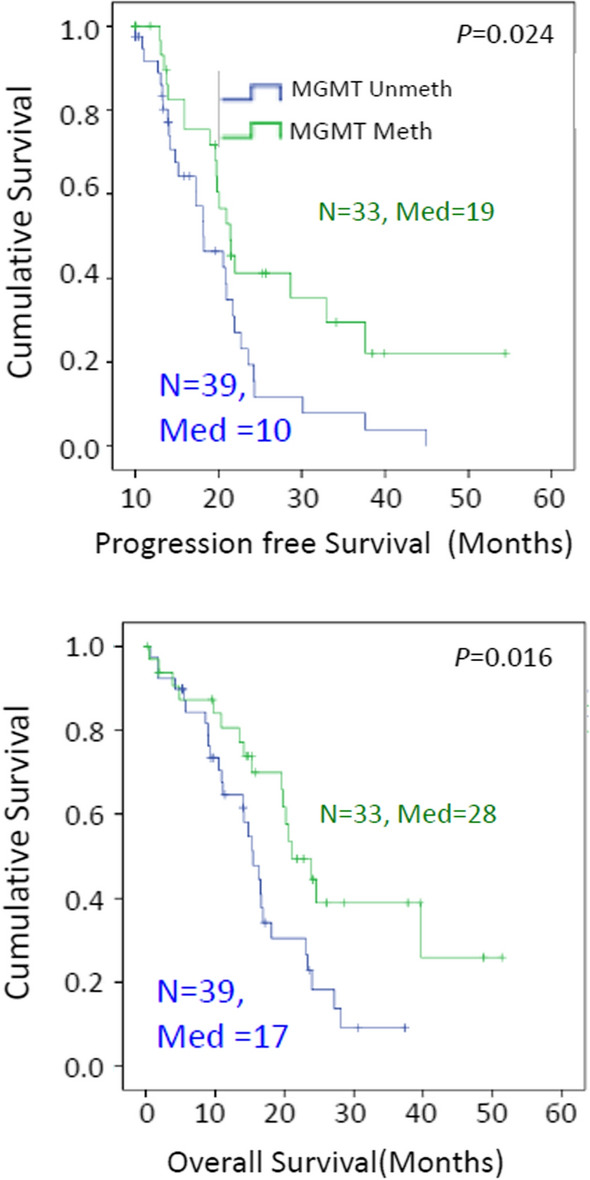
*MGMT* promoter methylation status is associated with both progression-free survival and overall survival in this cohort by Kaplan–Meier analysis. (Methylated, green; Unmethylated, blue, *N* = number of patients at time 0, Med = median survival)

As an initial evaluation of our cohort of 86 patients in the TMA group, characteristics previously associated with GBM patient groups were evaluated. The presence of *MGMT* promoter methylation was associated with both progression free and overall survival (Table 2 and Fig. 5), with patients whose tumors had methylated *MGMT* promoter surviving longer than patients whose tumors had unmethylated *MGMT* promoter, as described in prior studies. Age was also a significant predictor of overall survival in this cohort (*p* = 0.03 by Kaplan–Meier for categorical age < 62), but showed no statistically significant relationship with progression-free survival (*P* = 0.4). In this cohort, the survival for males was shorter than for females (Table 2).

**Table 2:** Cox proportional hazards ratio hazard ratio (HR) and *P*-value for each variable. *N* = 86, unless otherwise indicated

Characteristic	HR PFS	*P* PFS	HR OS	*P* OS
Univariate				
Age (continuous variable)	–	0.2	***1.03 (1.01–1.06)***	***0.02***
Age (Categorical variable: > or < 62)		0.4	***1.8 (1.1–3.1)***	***0.03***
MGMT promoter methylation status (n = 72)*	***0.5 (0.28–0.9)***	***0.02***	***0.47 (0.26–0.88)***	***0.02***
Gender	–	0.8	***0.56 (0.3–0.97)***	***0.04***
Resection	–	0.08	–	0.7
CD133	–	0.6	–	0.8
SOX2	–	1.0	–	0.9
NES Percentage	–	0.8	–	0.4
NES blood vessels	–	0.6	–	0.5
Multivariate				
Gender	1.2	0.7	0.6 (0.3–1.1)	0.1
Age (Categorical variable: > or < 62)	0.4	1.4	***1.9 (1.0–3.6)***	***0.05***
MGMT Promoter Methylation (*n* = 72)*	0.02	0.4 (0.2–0.9)	***0.5 (0.4–1.3)***	***0.04***

Significant results with *p*</=0.05 were intended to be bold and italicized

^*^ MGMT status unknown in 14 cases, and these cases are not included.

*HR* Hazard Ratio, *P* *p*-value, *PFS* Progressive free survival, *OS* Overall survival  *p* values at 0.05 or below are italicized and bold

SOX2 was located predominantly in the nucleus (Fig. 1a), with only faint cytoplasmic staining in most cases. Nuclear SOX2 staining was present with a score > 1 in 62% of the cases (Table 1). For CD133, the tumor was considered positive when the protein was localized to both the cytoplasmic membrane and the cytoplasm. Significant expression was observed in 35% of the cases (Table 1, Fig. 1b). SOX2 and CD133 expression levels were not significantly associated with each other; only 20 cases had both strong SOX2 and CD133 expression (*p* > 0.8). The lack of complete concordance between CD133 and SOX2 is consistent with variable phenotypic expression of individual stem cell markers. In this cohort, none of the stem cell markers (SOX2, CD133, nor NES) was associated with progression-free survival or overall survival in univariate analysis (Table 2). The strength of association between pairs of variables was assessed by Chi square analysis (Table 3). There were relationships (*p* < 0.05) between gender and *MGMT* promoter methylation status, and between gender and SOX2 expression level in this cohort (Table 3). These were the only statistically significant associations between the presence of putative CSC markers and clinicopathological variables.

**Table 3:** Strength of associations between clinicopathological variables.

Characteristic	SOX2	CD133	NES score	NES blood vessels*	Age (< 62)	Gender
MGMT Status (*N* = 72)	0.33	0.18	0.2	0.7	0.4	0.04
Resection	0.1	0.9	0.2	0.3	0.8	0.6
Gender	0.01	0.2	0.7	0.2	0.3	–
Age (< 62)	0.7	0.8	0.7	0.3	–	–
CD133	0.8	–	0.7	0.01	0.2	–
SOX2	–	0.2	0.4	0.005	0.9	–
NES Percentage	–	–	–	0.01	0.7	0.7

^*^ Fisher’s exact test

*P*-values were derived by Chi-square analysis. *N* = 86, unless otherwise indicated

**Fig. 6:**
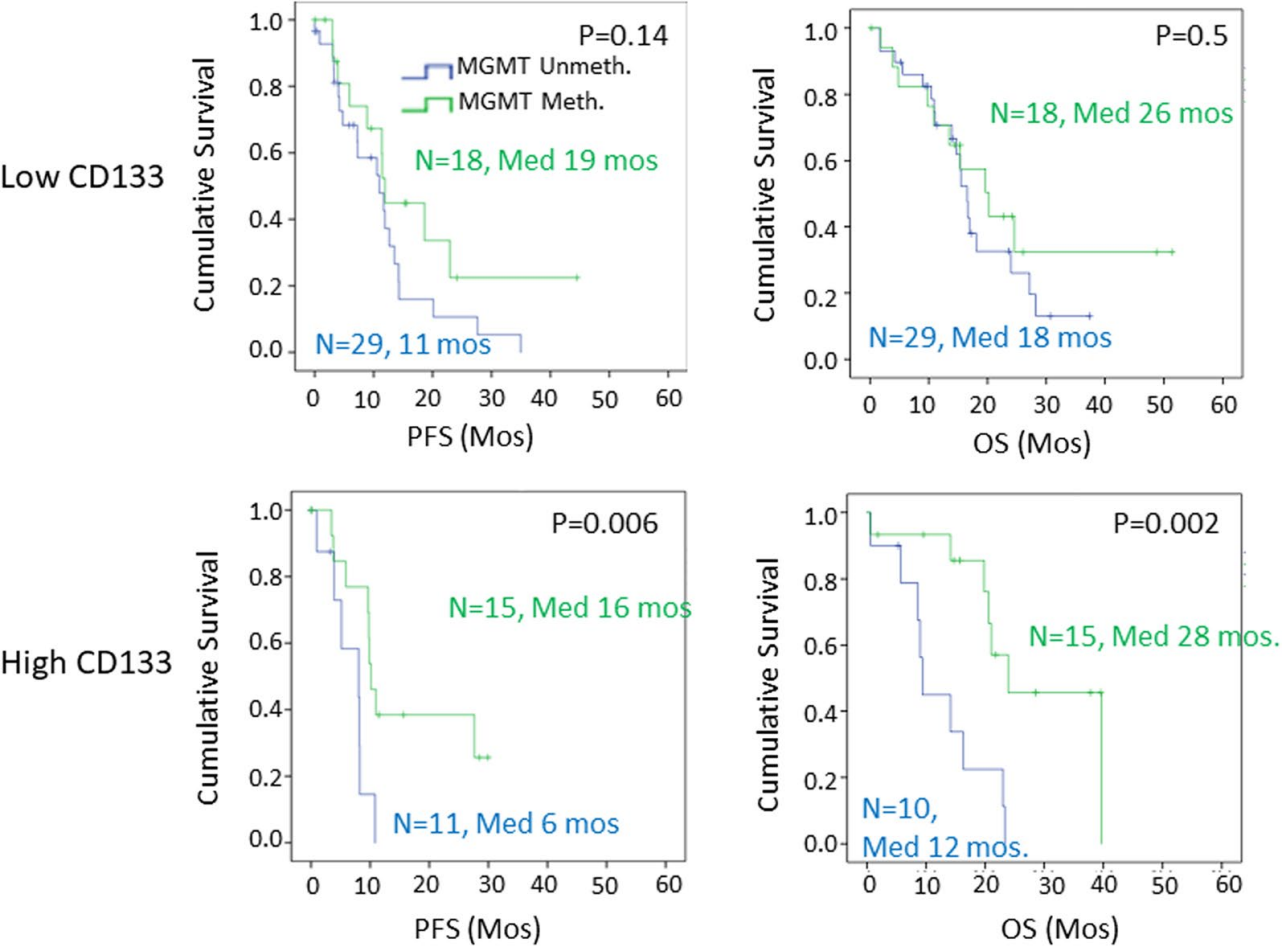
Stratification by CD133 expression reveals that *MGMT* promoter methylation is a predictor of progression-free survival (PFS) and overall survival (OS) by Kaplan–Meier analysis only in patients with high CD133 levels. In patients with low CD133 expression (raw score, product of intensity and percentage of cells, ≤ 100), there was no-significant difference in survival between those with methylated *MGMT* and those with unmethylated *MGMT*. (Methylated, green; Unmethylated, blue, *N* = number of patients at time 0, Median survival (Med) is in months; PFS-progression free survival, OS-overall survival)

In view of the hypothesis that the presence of stem cells within GBM may be related to chemoresistance and a quiescent phenotype within the tumor, we determined whether SOX2- or CD133-expression was associated with clinical outcome. Patients were stratified by their SOX2 or CD133 expression score, and *MGMT* promoter methylation status was evaluated as a risk factor by Kaplan–Meier analysis. In tumors with low CD133 expression (Score 0–1), *MGMT* promoter methylation did not distinguish high and low risk patient groups (Fig. 6). On the other hand, in tumors with high levels of CD133 expression, *MGMT* promoter methylation showed a strong significant relationship to both progression and overall survival (Fig. 6). The median overall survival for patients with high CD133 and unmethylated *MGMT* was 12 months, compared to 28 months in patients with high CD133 and methylated *MGMT* (Fig. 6; *p* = 0.002).

**Fig. 7:**
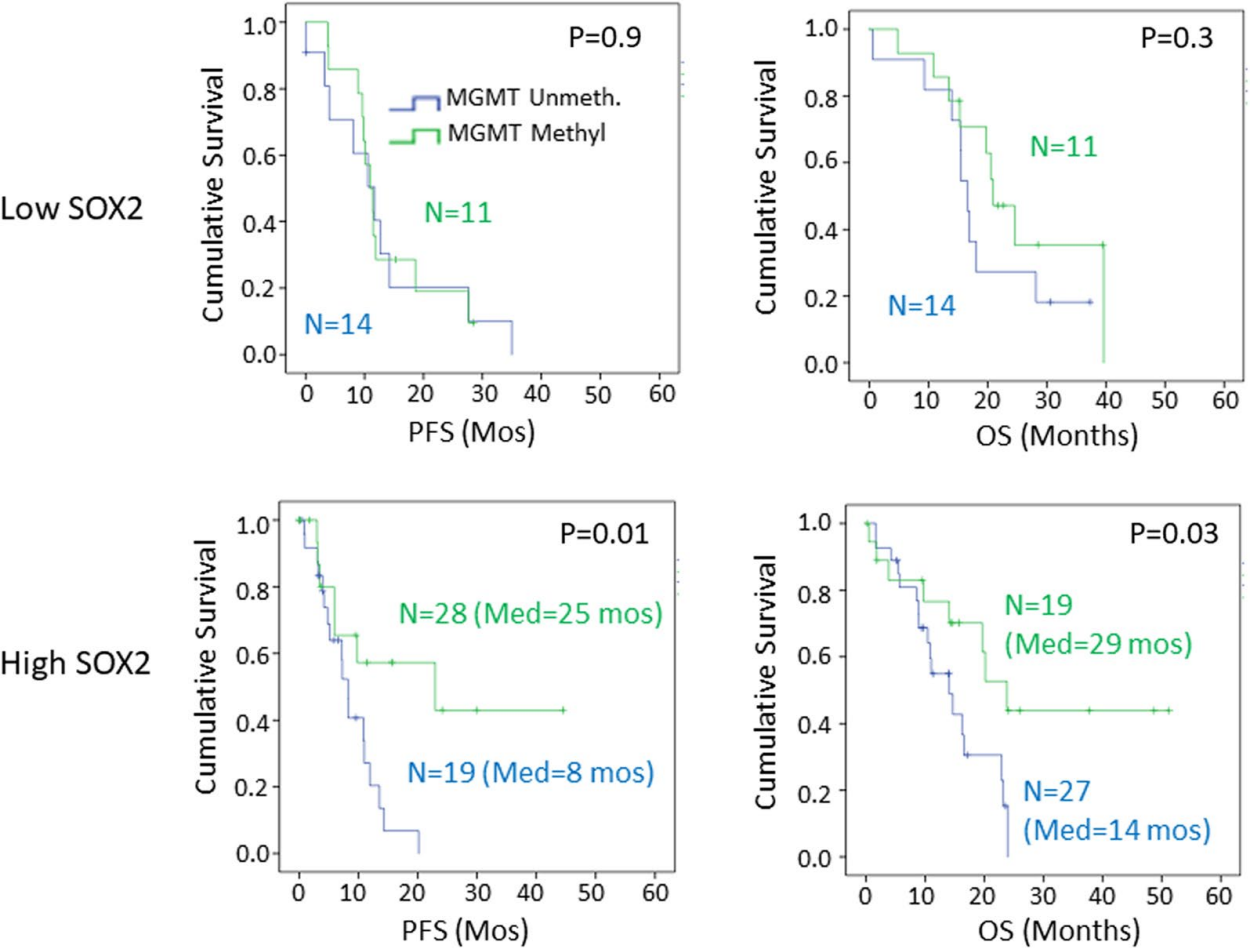
Stratification by SOX2 expression reveals that *MGMT* promoter methylation is a predictor of progression-free survival (PFS) and overall survival (OS) only in patients with high SOX2 (Raw score > 100) expression in Kaplan–Meier analysis. In patients with low SOX2 expression (Raw score ≤ 100), there was no-significant difference in survival between those with methylated *MGMT* and those with unmethylated *MGMT*. (Methylated, green, Unmethylated, blue *N* = number of patients at time 0, Med = median survival in months)

A similar relationship was seen for SOX2 expression (Fig. 7). Tumors with low SOX2 expression did not show significant differences in survivals, either overall survival or progression-free survival, based on *MGMT* promoter methylation status (Fig. 7). In contrast, *MGMT* promoter methylation strongly distinguished high and low risk patients in patients with high levels of SOX2 expression (Fig. 7). The median overall survival for patients with high SOX2 and unmethylated *MGMT* was 14 months, compared to 29 months in patients with high SOX2 and methylated *MGMT* (Fig. 7; *p* = 0.03). NES expression levels, which were high in both tumor cells and blood vessels in the great majority of tumors (Table 1), did not show any relationship with *MGMT* and survival.

**Fig. 8:**
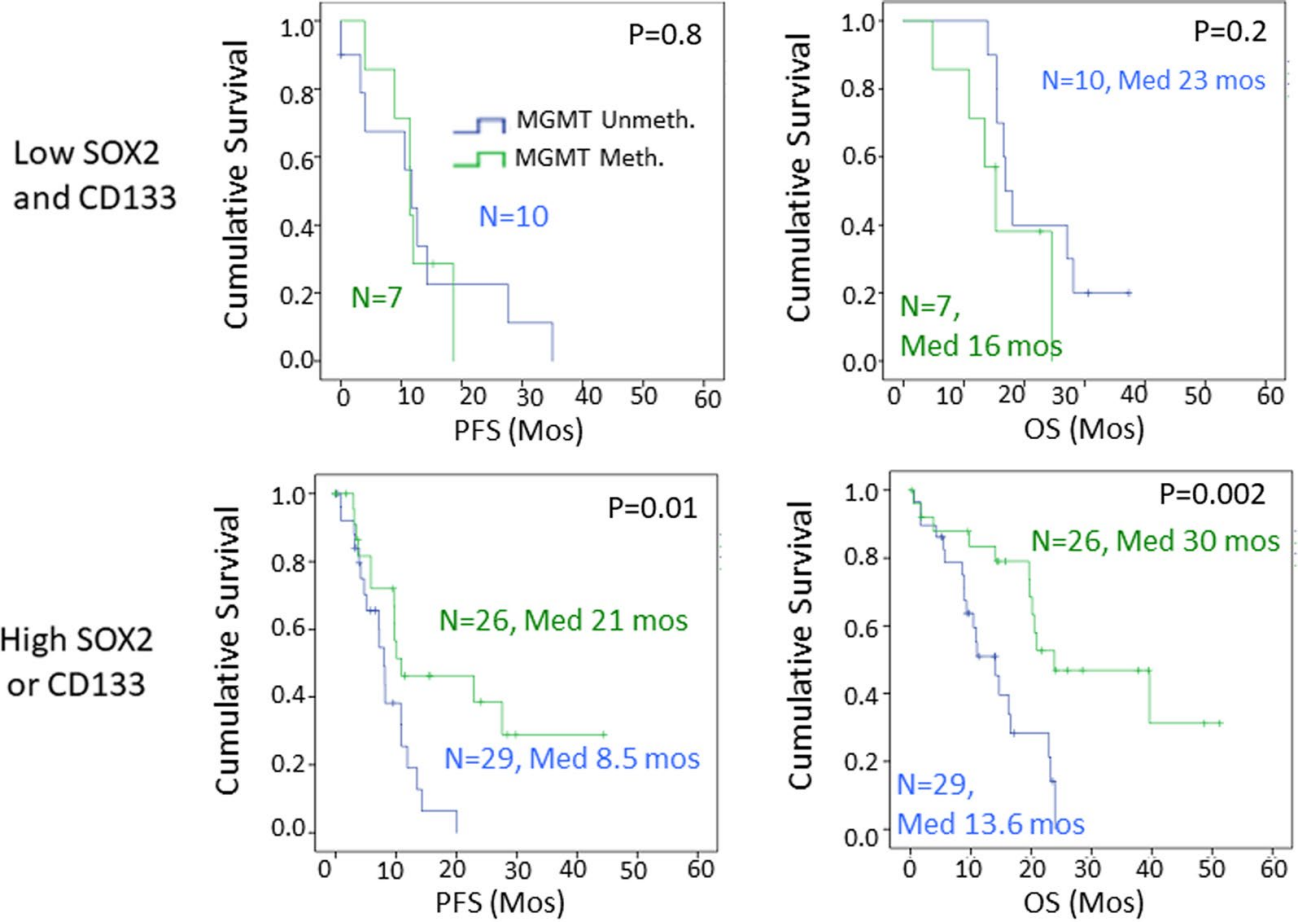
Stratification by the presence of either CD133 or SOX2 expression reveals that *MGMT* promoter methylation is a predictor of outcomes only with high level of expression of either CD133 or SOX2. Kaplan–Meier analysis. (Methylated, green; Unmethylated, blue, *N* = number of patients at time 0, Median survival (Med) is shown in months; PFS-progressive free survival, OS-overall survival)

Based on these findings, we grouped patients according to whether they had high expression of either CD133 or SOX2, versus low expression of both markers (Fig. 8). In this comparison, tumors with low stem cell markers showed a short overall survival and *MGMT* status was not predictive of survival in this group (Fig. 8). In contrast, for patients with tumors that had high levels of expression of either stem cell marker, methylated *MGMT* status was associated with longer survivals, both progression-free survival and overall survival (Fig. 8). The median overall survival for patients with high stem cell markers and unmethylated *MGMT* was 13.6 months, compared to 30 months in patients with high markers and methylated *MGMT* (Fig. 8; *p* = 0.002).

**Fig. 9:**
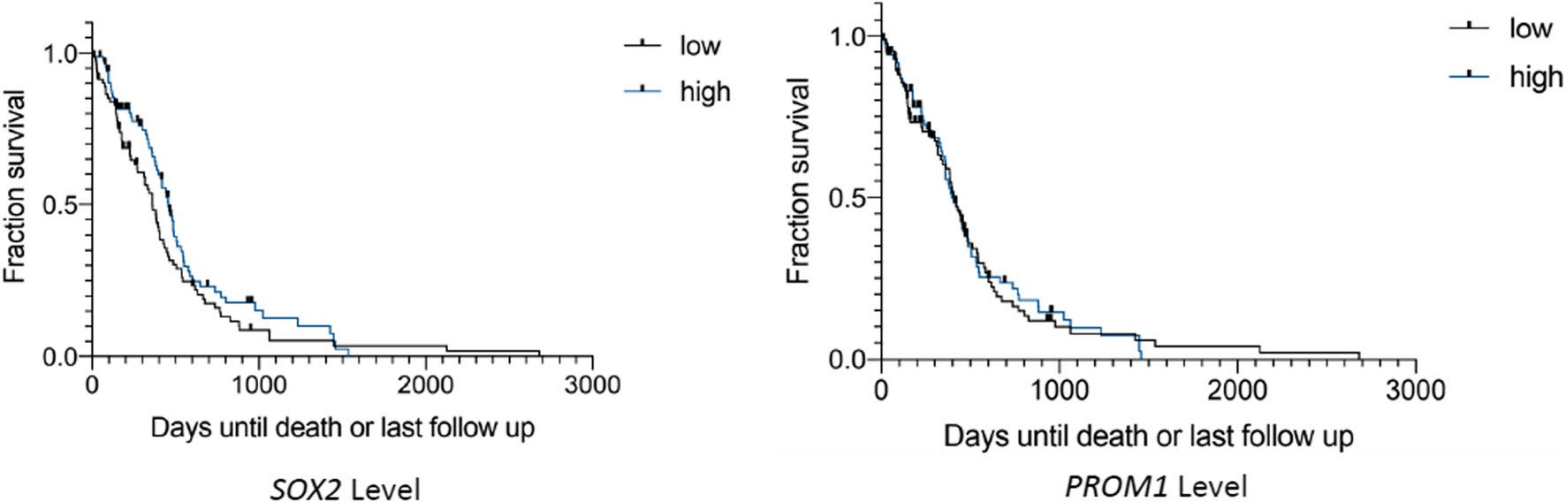
Kaplan–Meier survival curves for patients in the TCGA dataset, stratified by high and low expression (separated at the median) of *SOX2* (left) and *PROM1* (right). There is no significant difference in survivals based on these single gene expressions

**Fig. 10:**
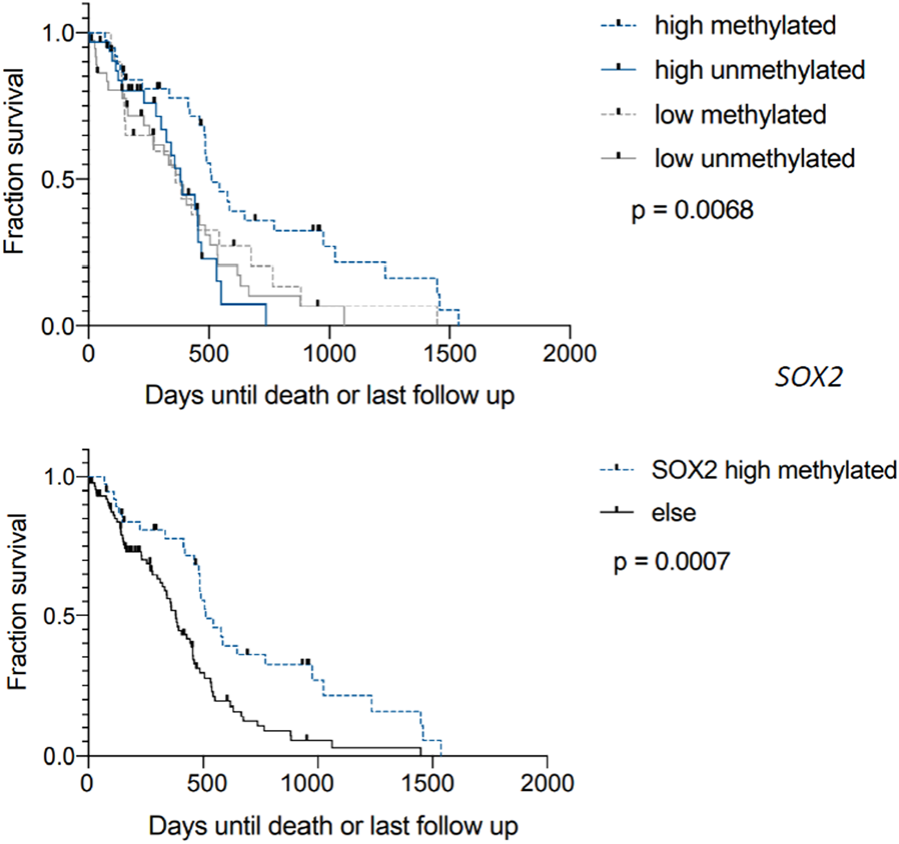
Kaplan–Meier survival curves for patients with GBM stratified by high or low *SOX2* (separated at the median level) and *MGMT* methylation status. The high *SOX2*/methylated group has the longest survival of the four groups (upper panel). Kaplan–Meier survival curves for patients with GBM comparing high *SOX2*/methylated patients to all other groups combined (lower panel)

**Fig. 11:**
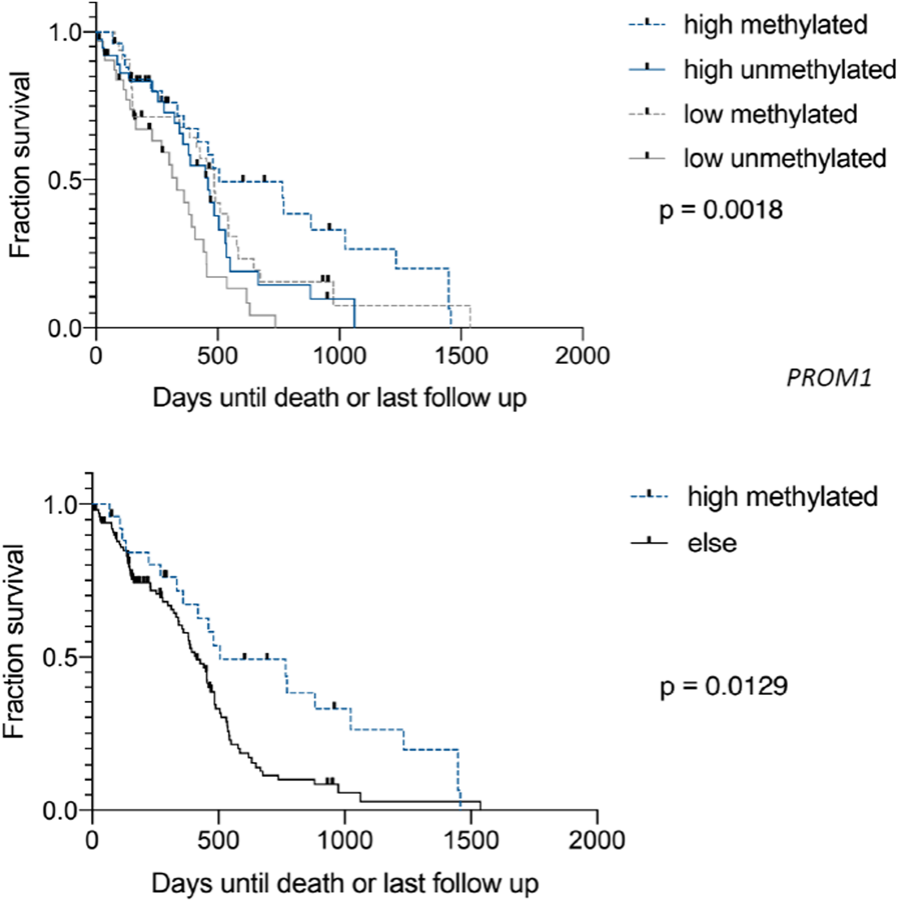
Kaplan–Meier survival curves for patients with GBM stratified by high or low *PROM1* (separated at the median level) and *MGMT* methylation status (upper panel). The high *PROM1*/methylated group has the longest survival of the four groups. Kaplan–Meier survival curves for patients with GBM comparing high *PROM1*/methylated patients to all other groups combined (lower panel)

**Fig. 12:**
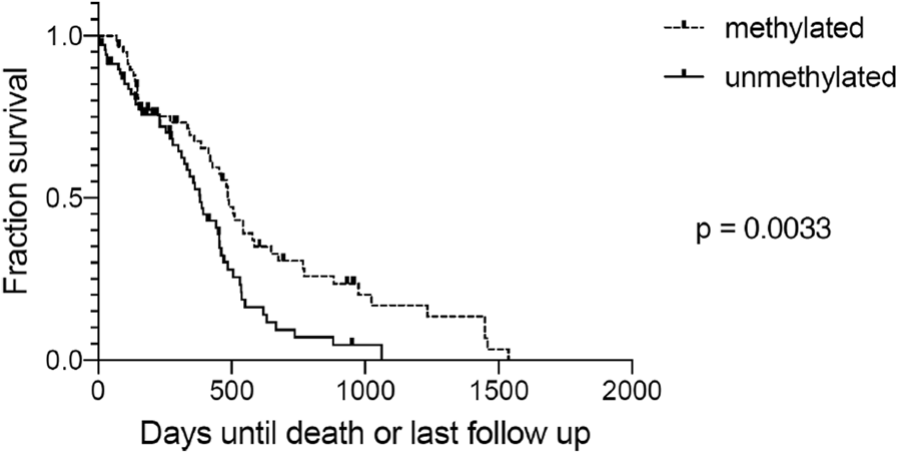
Kaplan–Meier survival curves for patients with GBM comparing *MGMT* methylated patients to MGMT unmethylated patients in the TCGA dataset

To evaluate these findings in a separate set of patients, we interrogated the TCGA dataset, separating cases by the median *SOX2* and *PROM1* (CD133) mRNA expression levels. Methylation status was available in the majority of TCGA cases, with similar distributions of methylated and unmethylated status in tumors with high and low *SOX2* and *PROM1* expression (Additional file 1). Kaplan–Meier survival curves from the TCGA dataset are shown in Figs. 9, 10, 11 and 12. When split by the median value, the level of *SOX2* expression did not correlate with survival, nor did the level of *PROM1* (Fig. 9). However, those patients in which the tumor had high *SOX2* expression and *MGMT* was methylated showed a longer survival than when unmethylated with high *SOX2* expression, or patients with low expression of *SOX2* regardless of *MGMT* methylation status (Fig. 10). When this group (high *SOX2* expression with *MGMT* methylated) was compared with all other patients combined (Fig. 10), median survival was extended nearly 5 months (*p* = 0.0007). Similarly, those patients in which the tumor had high *PROM1* expression and *MGMT* was methylated had a longer survival than unmethylated or patients with low expression of *PROM1*, regardless of *MGMT* methylation status (Fig. 11). When this group (high *PROM1* expression with *MGMT* methylated) was compared with all other patients combined (Fig. 11), median survival was nearly 2 months longer (*p* = 0.0129). As reported previously, methylation of MGMT was associated with increased survival in the TCGA dataset (Fig. 12, *p* = 0.0033).

## Discussion

The goal of this study was to evaluate the potential clinical significance of the presence of stem-like cells in glioblastomas. While a precise definition of cancer stem cells has not yet been established, there is substantial evidence that stem-like cells are present in GBM, that they share certain biologic characteristics with neural stem cells, and that they are associated with resistance to therapy [7, 9, 10, 13, 20, 23, 33, 34, 39]. This study was conducted to determine whether they also may have clinical significance in patients with GBM. In particular, we explored whether there were relationships between known prognostic factors and the presence of GBM cancer stem cells, as identified by cellular phenotypic markers. We hypothesized that the presence of a prominent population of cancer stem cells might predict the presence of drug resistance and may, therefore, be related to both progression-free and overall survival.

The abundance of stem cells was measured by three separate stem cell markers (CD133, SOX2, and NES) using semiquantitative multi-modal grading of immunohistochemistry and, in univariate analysis, did not show any relationship with progression-free survival or overall survival. In this cohort, CD133 was expressed in only 35% of cases, and we found no correlation between CD133 immunohistochemical expression and patient survival. SOX2 was observed in 94% of the cases, and it was highly expressed in 62% of cases (Score 2–3). By utilizing a modest and clearly defined scoring for SOX2 expression, we observed a large dynamic range of high (Score 2–3) and low (Score 0–1) SOX2 expression. Similarly, SOX2 expression level had no significant relationship with survival by univariate analysis (Table 2). The same was true for NES expression as an independent variable. Our data do not support a role for CSC’s as indicated by CD133, SOX2, or NES, as univariate predictors of outcome, and similarly we did not find a significant relationship between high *SOX2* or high *PROM1* expression and outcome in the TCGA dataset (Fig. 9).

In addition, there was no clear relationship between which tumors had high CD133 expression and SOX2 expression in our cohort (Table 3). SOX2 and CD133 (PROM1) are not correlated with each other in the Rembrandt and the TCGA datasets (Additional file 2). There was no significant relationship between *SOX2* and *MGMT* levels in TCGA and Rembrandt datasets (Additional file 3); *MGMT* methylation and SOX2 expression were not correlated in our patient dataset (Table 3).

Stemness can be defined in many different ways, leading to different observations reported in the literature. Recently, the PanCancer TCGA consortium derived a DNA methylation and RNA expression stemness index, but their association with outcomes was of borderline significance [23]. The RNA expression stemness signature includes both *SOX2* and *PROM1* (CD133), and is enriched in recurrent GBM [23]. In our study, we defined glioma stem cells by CD133 (PROM1) or SOX2 protein expression using semiquantative immunohistochemistry, recognizing that additional computational approaches may be more widely available in the near future.

When we studied whether stem cell markers are predictors of outcome in subsets of patients with GBM based on *MGMT* methylation status, we uncovered a remarkable relationship: *MGMT* methylation was a predictor of outcome only in tumors with strong levels of expression of SOX2 or CD133, and not in tumors with low or absent SOX2 and CD133 expression. A strong association between survival and *MGMT* methylation in the presence of a prominent stem cell component was present for CD133 alone, SOX2 alone, or the presence of either SOX2 or CD133. We tested this observation in the TCGA dataset, again finding that *MGMT* methylation status is associated with improved survival only in patients with high levels of *SOX2* or *PROM1*. Therefore, some of the variation in the prognostic impact of *MGMT* promoter methylation status may be related to the presence of treatment resistance associated with the stem cell phenotype. We propose that patients’ survival risk may be dependent on both the presence of *MGMT* promoter methylation and the stem cell level within the tumor.

It has been established that when *MGMT* methylation status was studied in patients treated with combined TMZ and radiation/surgery compared to radiation/surgery alone, the effect of methylation status is entirely related to TMZ treatment [17]. Because we find that this benefit of *MGMT* methylation is only observed in tumors with high levels of stem cell markers, it suggests that the impact of a high stem cell phenotype in GBM is entirely related to TMZ therapy. One possible explanation for this observation is that tumors with high levels of stem cells are more sensitive to TMZ therapy, while those with low expression of SOX2 or CD133 may not be as sensitive to this treatment. While there is limited in vitro evidence that stem cell phenotypes may be sensitive to TMZ [31], most ex vivo data suggests the opposite, that stem cells are resistant to TMZ therapy and adopt a quiescent phenotype [5, 13, 15, 20]. An alternative interpretation of our data is that TMZ treatment may induce quiescence in tumors that contain abundant stem cells at the onset of therapy. Our study did not address this question of therapy-induced quiescence, although recent studies of cellular phenotypes in recurrent GBM has suggested the emergence of a subpopulation of cells in GBM following therapy with some stem cell profiles, and that this cellular dormancy may be related to tumor mass dormancy in patients [1–3, 36], also suggesting the possibility of a temporary quiescence in GBM followed by later recurrence. Regardless of whether treatment initiates quiescence in GBM, in our dataset the longest survivals were observed in patients with high SOX2 or CD133 levels and methylated *MGMT*. This suggests that a critical effect of TMZ therapy in GBM may be dependent on the presence of stem cells, and has less effect in tumors with a low component of this cellular phenotype.

Our study did not determine which protein marker is the best for identification of a stem cell subpopulation; both CD133 and SOX2 showed statistically significant relationships with survival in tumors with methylated *MGMT*. As a practical matter, scoring of SOX2 is quantified more easily due to its clear nuclear localization, which is a benefit when using multi-modal quantitation in IHC. Extensive pre-clinical observations link CD133 with a population of progenitor, treatment resistant cells [5, 21], and increased expression of CD133 may be an indicator of cell stress. In our study, both markers of stem cell phenotype, CD133 and SOX2, affected the risk by stratifying patients into groups and evaluating their *MGMT* promoter methylation status.

Methylation of the *MGMT* promoter was associated with better outcomes when tumors expressed CD133 or SOX2 at high levels, but not in tumors with a lower level of stem cell phenotype. Our observations suggest that tumors with low levels of treatment resistant cancer stem cells all respond similarly to treatment, independent of *MGMT* promoter methylation status. On the other hand, our results suggest that tumors with significant stem cell populations may be more resistant to treatment, unless the *MGMT* promoter is methylated. The data also support that the combination of tumors with a high stem cell phenotype and unmethylated *MGMT* promoters had the most aggressive tumor progression and early death. As a result, patients with unmethylated *MGMT* promoter and high stem cell populations, and patients with low stem cell populations may benefit the most from alternative therapies.

We searched the literature to identify other datasets that could be used to compare with our observations. Pallini et al. evaluated 44 patients, all treated with TMZ, and evaluated the expression of Ki67, CD133, and *MGMT* relative to patient outcomes. They identified a combination of CD133 and Ki67 was associated with survival in 44 GBM cases [29]. However, the average overall survival of their cohort was only 13.3 months, and *MGMT* promoter methylation status was not associated with outcomes in their cohort [29]. After stratification by CD133 status, only 10 patients could be studied, and the numbers were too small to allow survival analysis. Melguizo et al*.* reported that CD133 levels were not associated with overall survival; only *MGMT* promoter methylation status was associated with both overall and progression free survival in their cohort [25].

In our cohort, age, male gender, and *MGMT* promoter methylation status were all associated with survival as has been seen in other cohorts. Multiple series have established the relationship between age and survival, and between *MGMT* methylation and survival. We also observed that male patients had significantly higher risk compared to females (Table 2). Male gender was only significant in our series in univariate analysis but not multivariate analysis (Table 2). The differences in outcomes between men and women have varied between studies; however, most studies show a survival advantage in women [27], similar to the results of our cohort. In particular, the survival advantage of women is true in IDH-wildtype patients, as in this study [27].

*MGMT* promoter methylation is currently used to assess response to TMZ therapy, and to date remains the single best predictor of response to combined therapy with TMZ and radiation/surgery. Age is also a well-established predictor of outcome in patients with glioblastoma. All major studies of outcomes have shown shorter survivals in patients at advanced age, especially those over the age of 65 [19]. We used both 65 years and 62 years (the median age in our cohort) as cut-offs, and our analyses showed a statistically significant difference in overall survival related to age. We did not observe any age dependent SOX2 correlations as has been reported in a smaller cohort [8]. *MGMT* expression level and *SOX2* expression level were weakly negatively correlated in the Rembrandt and TCGA datasets (Additional file 3).

## Conclusions

Our results show that the strength of *MGMT* promoter methylation as a predictor of survival is dependent on the abundance of cancer stem cells within the tumor. Both in our patient cohort and in the TCGA dataset cohort, MGMT methylation status failed to predict survival differences in patients with low levels of SOX2 or PROM1 (CD133), while there was a strong difference in survival based on MGMT methylation status in patients with high levels of SOX2 or PROM1 in their tumors. These findings suggest that tumors with abundant SOX2 or CD133 positive cancer stem cells at the time of diagnosis are more sensitive to current treatments when the *MGMT* promoter is methylated, warranting further investigation into the potential relationships between the degree of stem cell phenotype and response to therapy. As a result, it may be possible to eventually combine the presence of stem cell markers with *MGMT* promoter methylation status for improved prognostication for patients with IDH-wildtype GBM treated with TMZ and radiation/surgery. Although not directly associated with risk, SOX2 or CD133 in combination with other clinicopathological characteristics may be developed into useful predictors of sensitivity to TMZ or other therapies. Such a relationship has clear implications for targeted anti-stem cell therapies that might improve outcomes in patients with glioblastoma.

## Supplementary Information

**Additional file 1**. Number of patients in the TCGA dataset stratified by low and high SOX2 mRNA (A), or low and high PROM1 mRNA (B) by methylation status, where available.

**Additional file 2**. Lack of correlation of mRNA levels of PROM1 and SOX2 in GBM in the Rembrandt and TCGA datasets. TCGA Pearson r coefficient is 0.321.

**Additional file 3**. Lack of correlation between MGMT and SOX2 mRNA levels in the Rembrandt and TCGA datasets. TCGA Pearson r coefficient is -0.288.
