# Supplementary figures and images for: Stem cell phenotype predicts therapeutic response in glioblastomas with MGMT promoter methylation
Source: Acta Neuropathol Commun. 2022 Nov 4;10:159. doi: 10.1186/s40478-022-01459-9 (PMC9636755; doi:10.1186/s40478-022-01459-9)

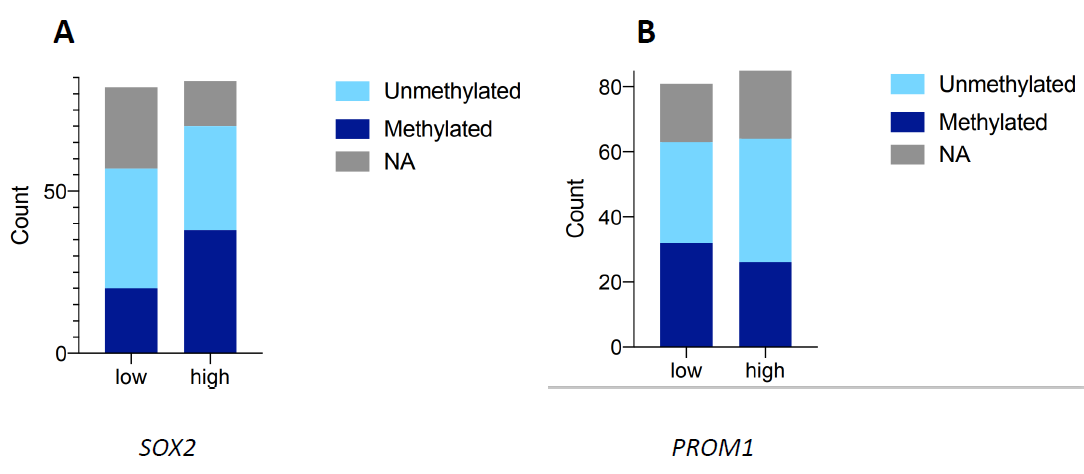

Supplement: Supplementary file 1 — Additional file 1. Number of patients in the TCGA dataset stratified by low and high SOX2 mRNA (A), or low and high PROM1 mRNA (B) by methylation status, where available. [file 40478_2022_1459_MOESM1_ESM.tif]

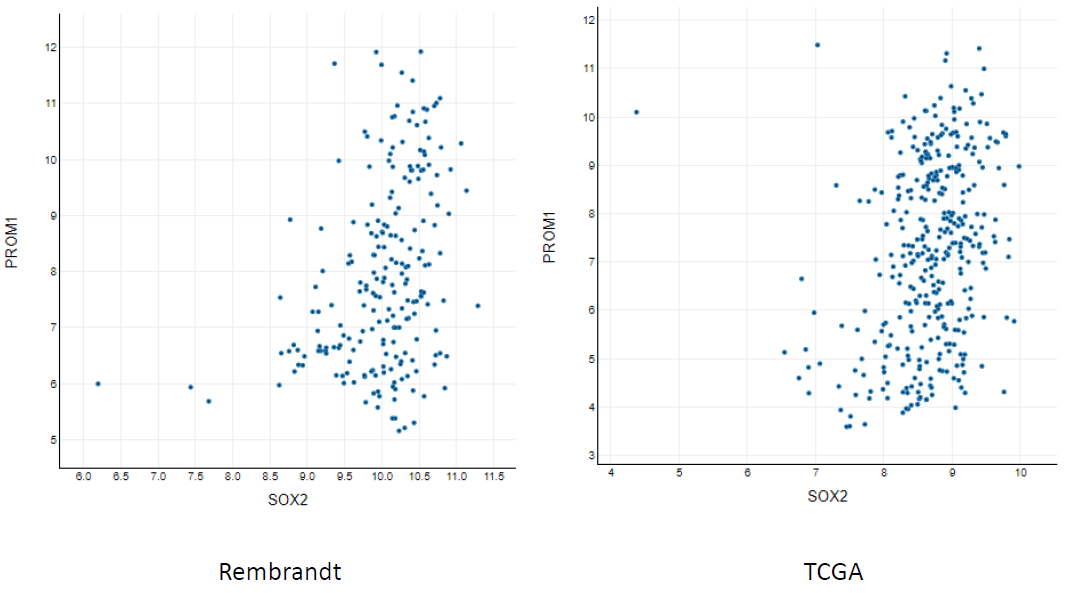

Supplement: Supplementary file 2 — Additional file 2. Lack of correlation of mRNA levels of PROM1 and SOX2 in GBM in the Rembrandt and TCGA datasets. TCGA Pearson r coefficient is 0.321. [file 40478_2022_1459_MOESM2_ESM.tif]

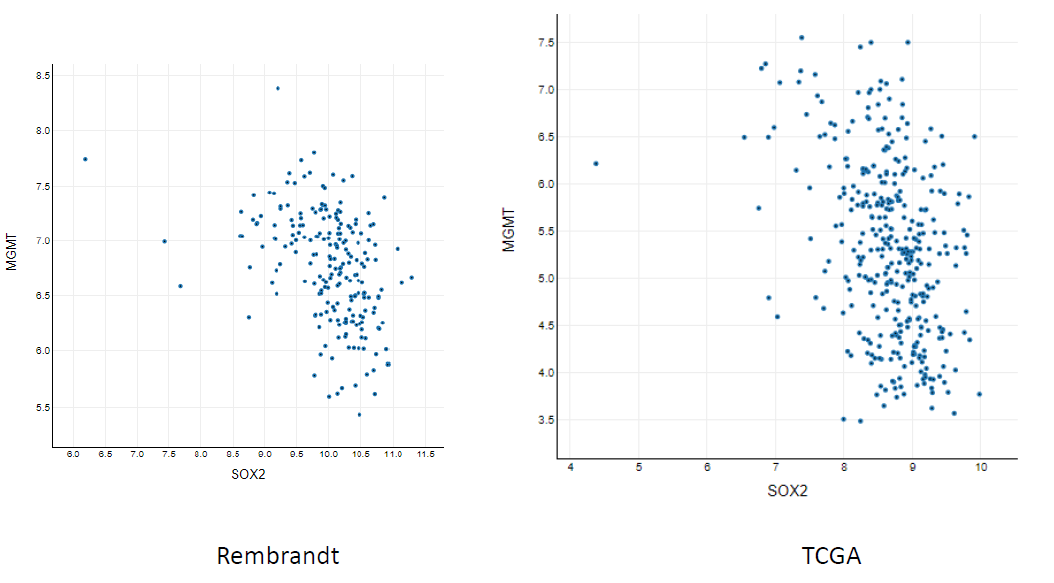

Supplement: Supplementary file 3 — Additional file 3. Lack of correlation between MGMT and SOX2 mRNA levels in the Rembrandt and TCGA datasets. TCGA Pearson r coefficient is -0.288. [file 40478_2022_1459_MOESM3_ESM.tif]
